# Targeting Sodium in Heart Failure

**DOI:** 10.3390/jpm14101064

**Published:** 2024-10-17

**Authors:** Filippos Triposkiadis, Andrew Xanthopoulos, John Skoularigis

**Affiliations:** 1 School of Medicine, European University Cyprus, 2404 Nicosia, Cyprus; 2 Department of Cardiology, University Hospital of Larissa, 41110 Larissa, Greece; anxanthopoulos@med.uth.gr (A.X.); iskoular@med.uth.gr (J.S.)

**Keywords:** sodium, hypertonic saline, congestion, sensors, urinary spot

## Abstract

A dominant event determining the course of heart failure (HF) includes the disruption of the delicate sodium (Na^+^) and water balance leading to (Na^+^) and water retention and edema formation. Although incomplete decongestion adversely affects outcomes, it is unknown whether interventions directly targeting (Na^+^), such as strict dietary (Na^+^) restriction, intravenous hypertonic saline, and diuretics, reverse this effect. As a result, it is imperative to implement (Na^+^)-targeting interventions in selected HF patients with established congestion on top of quadruple therapy with angiotensin receptor neprilysin inhibitor, β-adrenergic receptor blocker, mineralocorticoid receptor antagonist, and sodium glucose cotransporter 2 inhibitor, which dramatically improves outcomes. The limited effectiveness of (Na^+^)-targeting treatments may be partly due to the fact that the current metrics of HF severity have a limited capacity of foreseeing and averting episodes of congestion and guiding (Na^+^)-targeting treatments, which often leads to dysnatremias, adversely affecting outcomes. Recent evidence suggests that spot urinary sodium measurements may be used as a guide to monitor (Na^+^)-targeting interventions both in chronic and acute HF. Further, the classical (2)-compartment model of (Na^+^) storage has been displaced by the (3)-compartment model emphasizing the non-osmotic accumulation of (Na^+^), chiefly in the skin. 23(Na^+^) magnetic resonance imaging (MRI) enables the accurate and reliable quantification of tissue (Na^+^). Another promising approach enabling tissue (Na^+^) monitoring is based on wearable devices employing ion-selective electrodes for electrolyte detection, including (Na^+^) and (Cl^–^). Undoubtably, further studies using 23(Na^+^)-MRI technology and wearable sensors are required to learn more about the clinical significance of tissue (Na^+^) storage and (Na^+^)-related mechanisms of morbidity and mortality in HF.

## 1. Introduction

Chronic heart failure (HF) is a rapidly expanding public health issue, which currently affects approximately 64 million people worldwide [1]. Despite the significant improvements in medical and device management, many HF patients have a precarious prognosis [2]. Dyspnea and edema in their various expressions are among the major symptoms of HF patients, progressing relentlessly with disease advancement and eventually leading to severe limitations in the capacity to function and accomplish the tasks of everyday life in the advanced disease stage [3]. Both dyspnea and edema in HF develop in the setting of congestion, defined as sodium (Na^+^) and fluid accumulation, resulting from maladaptive (Na^+^) and water retention, predominantly in the kidneys [4].

(Na^+^) is the pre-eminent cation of the extracellular fluid (ECF), which consists of blood, lymph, and cerebrospinal fluid, and, together with the associated anions, contributes to 90% of ECF osmolality [5]. The ECF, containing approximately 1/3 of the total body fluid, has a (Na^+^) concentration ≈ 144 mOsm/L, while the intracellular fluid (ICF) has a much lower (Na^+^) concentration (≈10 mOsm/L) [6]. Considering the stability of water and (Na^+^) levels is crucial for proper cellular function and the solute permeability of the cell membrane is low, water movement is osmotically driven from areas of lower to those of higher solute concentrations, aiming at the equalization of osmotic ECF and ICF pressures. The preservation of osmolality requires that, after a rise in salt (NaCl) in a diet, each ≈140 mmol of additional (Na^+^) accumulated in the extracellular space must be linked with the amassment of ≈1 L of water in the ECF [6]. Thus, (Na^+^) is foundational for maintaining the balance of electrolytes and water (hydromineral regulation) [7].

Patients with HF often develop severe congestion necessitating hospital management, especially if it is predominantly present in the lungs [8,9]. Intuitively, dietary (Na^+^) restriction often accompanied by diuretic treatment is usually recommended by clinicians to HF patients [10,11]. Regarding diuretic use, the National Institute of Health and Care Excellence (NICE) guidelines recommend using diuretics to improve symptoms [12], while the American Heart Association (AHA) guidelines recommend the use of diuretics to improve symptoms and prognosis [11]. However, in the modern era of neurohormonal inhibition in HF, there are limited data to support this position [13].

In this manuscript, after a short discussion of the (Na^+^) balance and the mechanisms of (Na^+^) abundance and storage in HF, we summarize the effects of HF treatments directly targeting (Na^+^) balance (dietary (Na^+^) restriction, intravenous hypertonic saline [IHS], and loop diuretics) and touch upon the application of these interventions in daily practice.

## 2. Sodium Balance

Body (Na^+^) levels must be maintained within a narrow range for the correct functioning of organism (Na^+^) homeostasis. The balance in body (Na^+^) levels requires a delicate equilibrium to be maintained between the ingestion (gut absorption) and excretion (urine, feces, and skin) of (Na^+^) [14]. Homeostatic osmoregulation by (Na^+^), which is usually consumed together with chloride in the form of NaCl, is vital for life because severe hyper- or hypotonicity elicits irreversible organ damage and lethal neurological trauma.

Two mechanisms are predominantly responsible for the (Na^+^) balance and hydromineral homeostasis [14,15]. The first is determined by the intake of NaCl, which accumulates in the extracellular space and displaces water from the intracellular to extracellular space, causing cell dehydration. In this setting, the appetite for (Na^+^) is inhibited, renal (Na^+^) excretion is increased (natriuresis), water intake is enhanced, and fluid excretion is reduced (antidiuresis). Conversely, a reduction in ECF osmolality/natremia inhibits water intake, promotes diuresis, increases the appetite for (Na^+^), and suppresses natriuresis. The second mechanism is activated in response to loss of intravascular fluid (hypovolemia), typically caused by diarrhea, vomiting, hemorrhages, sweating, and renal or cardiovascular disorders. Hypovolemia triggers compensatory behavioral and physiological reactions to restore (Na^+^) and body fluids, including thirst and appetite for (Na^+^), especially for drinks containing the same NaCl concentration as intravascular fluid (0.15 M), as well as antidiuretic and antinatriuretic responses to retain water and (Na^+^). Conversely, ECF hypervolemia reduces the intake of water and (Na^+^) and increases diuresis and natriuresis.

Dysnatremias, namely hyponatremia and its opposite hypernatremia defined by the presence of a serum (Na^+^) concentration < 135 mEq/L and >145 mEq/L, respectively, are the most common electrolyte disorders encountered in hospitalized patients [16,17]. Hyponatremic plasma causes the intracellular movement of water, leading to cellular swelling, whereas hypertonic plasma causes the extracellular movement of water begetting cellular shrinkage. Despite the fact that total-body NaCl content may be abnormal, most dysnatremias are due to (Na^+^)-free water imbalance and their accurate classification and consequent treatment requires the use of formulas [18,19]. It is noteworthy that dysnatremias occur more commonly in the elderly, and their prevalence exhibits seasonal dependence, with hyponatremia being more frequent in the summer [20] and hypernatremia in winter [21].

## 3. Sodium Tissue Storage in Heart Failure

Body (Na^+^) is significantly expanded in HF due to the augmented (Na^+^) avidity of the kidney, and less so of the gut, triggered by a myocardial injury, leading to arterial underfilling and the overactivity of neurohormonal systems, the most important being the sympathetic nervous system (SNS) and the renin–angiotensin–aldosterone system (RAAS) [22,23,24,25,26,27].

According to the (2)-compartment model, conceived by Borst [28] and later refined by Guyton [29], the everlasting regulation of arterial pressure is coupled with the homeostasis of body fluid. In this regard, a rise in extracellular (Na^+^) due to high (Na^+^) intake or hypertonic NaCl infusion will raise extracellular osmolality and induce a water movement from the intracellular to the extracellular compartment aiming at the restoration of plasma osmolality. Further, this rise in plasma osmolality will generate thirst, leading to increased water intake and induce renal water retention in response to antidiuretic hormone (ADH) secretion. Owing to the subsequent rise in total body water, plasma osmolality will be restored associated by an expansion of ECF volume and elevation in blood pressure, which, subsequently, will initiate pressure natriuresis and decrease total body (Na^+^) content.

**Figure 1:**
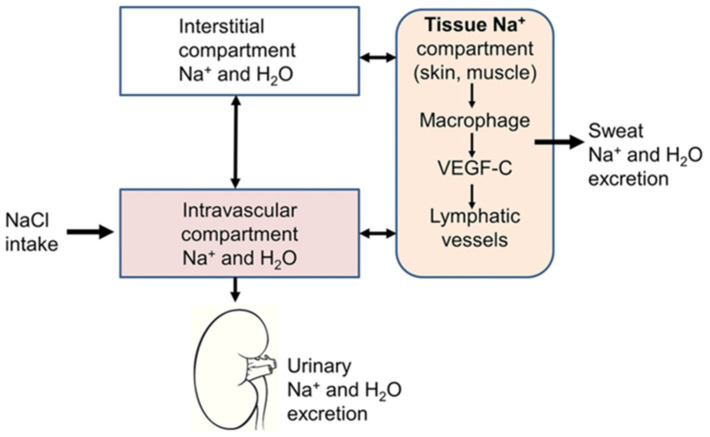
The (3)-compartment model. Sodium is stored in tissues (e.g., skin or muscles) in addition to the intravascular and interstitial compartments. The third compartment sodium is osmotically inactive and can be either returned to the intravascular compartment through lymphatic vessels or excreted through the sweat. (This figure is adapted from Ref. [30]. Polychronopoulou E, Braconnier P, and Burnier M (2019) *New Insights on the Role of Sodium in the Physiological Regulation of Blood Pressure and Development of Hypertension*. Front. Cardiovasc. Med. 6:136.)

The (2)-compartment model has been challenged by observations indicating that (Na^+^) can be stored in various body tissues (e.g., skin, muscle, and possibly the blood vessel wall) in levels exceeding by far those in plasma forming a third dynamic compartment to be added to the classical (2)-compartment model ((3)-compartment model) (Figure 1) [30]. In this regard, the Mars 500 study, evaluating (Na^+^) handling at stable NaCl intake for nearly long time periods, demonstrated that (Na^+^) is neatly accumulated and secreted independent of NaCl intake and that blood pressure, body weight, and extracellular water were poorly related to urine (Na^+^) excretion, indicating that part of the digested (Na^+^) is non-osmotically stored [31]. In the same direction, the findings of a study investigating the effect of intravenous hypertonic saline (IHS) infusion on (Na^+^) and water excretion in healthy humans reported that (Na^+^) recovery in urine was ≈50% of the anticipated [32]. Likewise, a study subjecting participants to ^23^(Na^+^) magnetic resonance imaging (^23^(Na^+^)-MRI) in order to quantify (Na^+^) content in skeletal muscle and skin reported a 29% increase in (Na^+^) content in patients with aldosteronism than those without, which was mobilized without weight loss after successful treatment [33]. Finally, a study including patients with HF (*n* = 18), in hemodialysis (*n* = 34), and chronic kidney disease (*n* = 31), who underwent ^23^(Na^+^)-MRI of the calf to quantify tissue (Na^+^), reported that those with HF had very high levels of skin (Na^+^), comparable to those of patients with end-stage kidney disease requiring renal replacement therapy (RRT) with hemodialysis [34].

**Figure 2:**
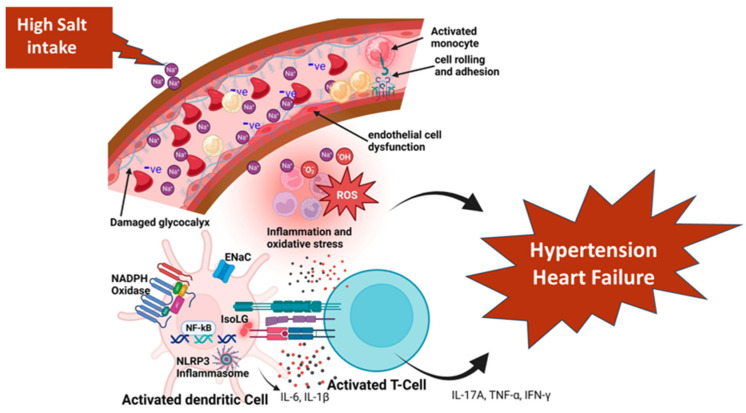
Mechanisms of damage to the glycocalyx induced by salt, resulting in hypertension and cardiovascular disease. High salt intake impairs glycocalyx and induces inflammation, oxidative stress, and immune activation, leading to the development of hypertension and cardiovascular disease. ROS, reactive oxygen species; ENaC, epithelial sodium channel; NADPH, reduced nicotinamide adenine dinucleotide phosphate; NLRP3, NLR Family Pyrin Domain Containing 3; NF-Kb, Nuclear factor kappa-light-chain-enhancer of activated B cells; IsoLGs, Islovuglandins; TNF-α, tumor necrosis factor alpha; IFN-γ, Interferon gamma. (This figure is adapted from Ref. [42]. Sembajwe, L.F.; Ssekandi, A.M.; Namaganda, A.; Muwonge, H.; Kasolo, J.N.; Kalyesubula, R.; Nakimuli, A.; Naome, M.; Patel, K.P.; Masenga, S.K.; et al. *Glycocalyx–Sodium Interaction in Vascular Endothelium*. Nutrients 2023, 15, 2873).

The skin, the largest human organ amounting 6% of body weight, is a significant part of the interstitial space (ISS) [35,36] and contains ten times more nitric oxide (NO) than the circulation which is an important modulator of the vascular tone [37]. Skin blood flow exhibits wide variations, having a minimum of ≈1% in low temperatures and a maximum of ≈60% in erythroderma and heat stress, indicating its capacity to regulate systemic blood pressure [38,39]. This is concordant with the fact that the skin is a depot for (Na^+^), chloride, and water, despite the fact that the physiological significance of skin electrolytes has not been entirely delineated [6]. The accumulation of (Na^+^) in the skin is accompanied by an increase in the concentration and sulfation of negatively charged glycosaminoglycans (GAGs) [6], which facilitate the non-osmotic (Na^+^) storage in the ISS [40]. GAG polymerization enables osmotically inactive (Na^+^) accumulation in the skin, with skin (Na^+^) concentrations rising up to 180–190 mmol/L in the absence of commensurate elevations in the skin water content. These support the premise that the skin represents a “third compartment” of body (Na^+^), having a dynamic capacity for (Na^+^) storage via the stimulation of GAG synthesis, thus buffering the changes in blood volume and pressure with NaCl intake [41]. Equally important is the fact that GAGs are also a major part of the endothelial glycocalyx (eGC; a jelly-like protective layer covering the luminal surface of the endothelium), with damage by excessive NaCl intake that increases vascular permeability, leading to ISS fluid shifts, edema, and tissue (Na^+^) accumulation (Figure 2) [42].

Skin (Na^+^) may also have several important physiological functions relevant to HF development. Skin (Na^+^) gradients may generate a hypertonic barrier regulating fluid loss, similar to that of the countercurrent mechanisms in the kidney [43]. Further, it has been postulated that the skin (Na^+^) augments microbial host defense through macrophage-induced elevations in NO contributing to microorganism clearance [44]. However, it is currently unclear whether excess (Na^+^) in the skin prevents (Na^+^) overflow into the systemic circulation and, therefore, the development of cardiovascular disease, or whether it represents an overflow reservoir once the (Na^+^) excess has already caused damage to the vasculature enabling (Na^+^) leakage into the nearby tissue [41]. Moreover, there is evidence suggesting that high levels of extracellular (Na^+^) may trigger an inflammatory reaction involving antigen-presenting cells (APCs), which subsequently leads to hypertension and vascular and renal injury. It is noteworthy that (Na^+^) entrance into APCs is arbitrated by ENaC, suggesting that this channel may also contribute to blood pressure modulation involving extrarenal tissues through an immune-dependent mechanisms [45]. Further, gut microbiota, which constantly interact with the immune system and is crucial for the appropriate maturation process of immune cells, contribute to the inflammatory response to (Na^+^) as high-(Na^+^) diets promote the local and widespread inflammation of tissues and harm intestinal morphology compared with low (Na^+^) intake [46].

Skin (Na^+^) abundance in HF can be addressed with current medical treatment. In a study including patients with acute HF, ^23^(Na^+^)-MRI was performed prior to and following diuretic treatment to quantitate the (Na^+^) content in the lower leg muscle and skin [47]. Plasma (Na^+^) levels were unaffected by therapy. In contrast, (Na^+^) levels in muscle and skin decreased after treatment with furosemide. Furosemide therapy did not decrease water content in the muscle and tended to reduce water in the skin. Likewise, in another study including chronic HF patients treated with empagliflozin and subjected to ^23^(Na^+^)-MRI) at baseline, and after 1 and 3 months of treatment, a significant decrease in skin (Na^+^) content was observed, while there was no change in muscle (Na^+^) and muscle water content [48].

In summary, non-osmotic (Na^+^) storage in the skin seems to function as a double-edged sword. On one hand, it may be protective by buffering the adverse effects of excess (Na^+^), contributing, therefore, to the maintenance of (Na^+^) homeostasis. On the other hand, this useful buffering system may be overwhelmed by (Na^+^) leakage accompanied by damage to the eGC barrier and infiltration with inflammatory cells, eventually increasing cardiovascular risk. Whether skin (Na^+^) content should be considered a treatment target deserves further investigation.

## 4. Interventions Directly Affecting Body Sodium and Water in Heart Failure

### 4.1. Dietary Sodium Restriction

Currently, global NaCl intake, which is a major source of (Na^+^), amounts to 6.75–10.66 g/day [49], which is considerably higher than the recommended levels by the World Health Organization ([<2 g/day (Na^+^); 5 g/day NaCl)] in order to reduce blood pressure and cardiovascular risk in adults [50]. Dietary (Na^+^) restriction for HF patients is based on the premise that (Na^+^) intake leads to NaCl and water retention, which adversely affects outcomes. However, it is difficult for most patients to adhere to diets severely restrictive in NaCl and fluid intake [51]. Moreover, evidence supporting the implementation of (Na^+^) or fluid restriction is limited. Clinical studies associating (Na^+^) intake with HF outcomes in populations considered high risk, such as right-sided HF, hypertensive HF, and patients with high utilization of loop diuretics, remain limited and inconsistent [52].

High-quality evidence on the amount of (Na^+^) that a HF patient should consume originates from the SODIUM-HF trial, a multicenter, international, randomized, controlled trial (RCT), in which HF patients were randomly assigned to dietary (Na^+^) restriction < 1500 mg daily or usual care devoid of dietary (Na^+^) restriction [53]. Median dietary (Na^+^) intake at baseline was ≈2200 mg/day in all patients and was reduced by ≈415 mg/day in the low-(Na^+^) group. The composite primary outcome (total mortality, cardiovascular hospitalizations, or emergency department visits due to cardiovascular causes) did not significantly differ between the two groups of the trial at 12 months. Notwithstanding the fact that some improvement in life quality and NYHA functional class was observed, the possibility of reporting biases influencing outcomes, particularly in the low (Na^+^) group, cannot be excluded as SODIUM-HF was an open-label trial.

Towards the same direction were the findings of two recently published meta-analyses examining the impact of dietary (Na^+^) restriction in HF patients. The first, which included nine interventional and observational studies, reported that (Na^+^) restriction in chronic HF patients had an insignificant effect on all-cause mortality and HF hospitalization (HFHJ risk [54]. The second meta-analysis, which included 17 RCTs, also reported that (Na^+^) did not reduce all-cause mortality, HFH, or the composite of death/hospitalization, and that among the trials including Health-Related Quality of Life (HRQoL) as an endpoint, 6 showed HRQoL improvement with (Na^+^) restriction, whereas 4 reported an insignificant effect [55]. It is noteworthy that among the studies of this meta-analysis, in those with a NaCl intake 2000–3000 mg/day, the risk of all-cause mortality was lower compared to that in studies with a NaCl intake < 2000 mg/day, suggesting a varying effect related to the intensity of (Na^+^) restriction [55].

### 4.2. Intravenous Hypertonic Saline

**Figure 3:**
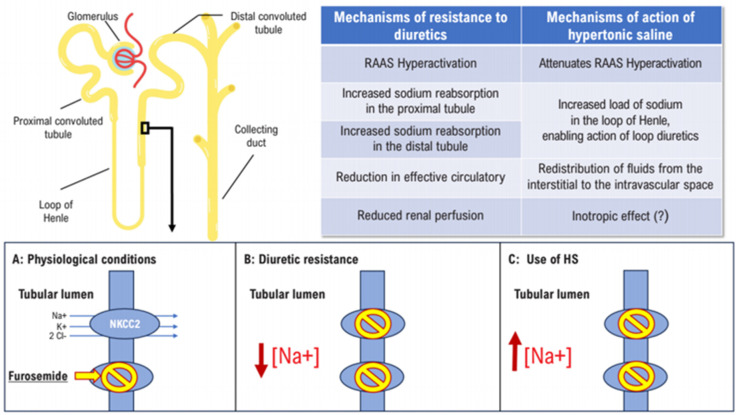
Mechanisms of diuretic resistance and hypertonic saline (HS). The lower panel depicts the apical membrane of the tubular cells of the thick ascending limb of the Henle loop (**A**) under physiological conditions, the Na^+^/K^+^/Cl^−^ cotransporter 2 (NKCC2), which is blocked by loop diuretics, contributes to the reabsorption of up to 25% of filtered sodium. (**B**) In cases with diuretic resistance, sodium reabsorption increases in the different segments of the nephron, resulting in lower concentration of sodium in the tubular lumen of the Henle loop and, therefore, less sodium excretion in urine and less diuresis. (**C**) With the use of HS, sodium concentration increases in the tubular lumen, potentiating the action of loop diuretics and attenuating diuretic resistance. HS: hypertonic saline. (This figure is adapted from Ref. [58]. *Hypertonic Saline Solution: How, Why, and for Whom?* Ciro Mancilha Murad1 and Fabiana Goulart Marcondes-Braga. ABC Heart Fail Cardiomyop. 2023; 3(2):e20230078).

The administration of IHS in HF seems counterintuitive, given the concept that (Na^+^) has been considered harmful for these patients. The main arguments underlying the combined use of IHS and furosemide in HF are that IHS (a) creates an osmotic gradient that drives fluid from the ISS into the intravascular compartment [56], (b) expands plasma volume changes, which increases the transport of water and (Na^+^) in the distal nephron [57], and (c) potentially attenuates RAAS overactivity and supplies (Na^+^) to the renal tubules, potentiating the action of loop diuretics (Figure 3) [58].

A retrospective analysis at a large US center demonstrated that IHS on top of loop diuretics was safe and well tolerated in severely ill patients with high serum creatinine, hyponatremia, and stagnant urine output in the 72 h prior to therapy implementation [59]. IHS administration was associated with a trend towards an increase in urine output, weight loss, and diuretic efficiency, improvement in renal function, and restoration of electrolyte abnormalities, raising the possibility that IHS may be effective in selective HF patients with refractory volume overload [59]. A recent meta-analysis including 10 RCTs involving 3013 patients with acutely decompensated HF reported that IHS on top of furosemide significantly reduced hospital stay, weight, serum creatinine, and type-B natriuretic peptide compared with furosemide monotherapy [60]. Further, IHS on top of furosemide significantly increased urine output, serum (Na^+^), and urine (Na^+^) compared with furosemide monotherapy.

In contrast to previous studies including hospitalized HF patients, a recent double-blind study, which evaluated the effect of a single infusion of IV furosemide with IHS in ambulatory patients with worsening HF, did not improve diuresis, natriuresis, or other congestion parameters in the short term in comparison with IV furosemide alone in ambulatory patients with worsening HF [61]. Although, in this study, treatment with IV furosemide–IHS led to a significant weight loss at 7 days, this outcome may have been confounded by several factors, further raising uncertainty whether IV IHS–furosemide may have any additional benefit in the short term over most traditional ambulatory diuretic approaches in outpatients with worsening HF.

Based on the above, the possibility of IV IHS–furosemide having additional benefits in the short term over most traditional diuretic approaches in HF cannot be excluded. The conduction of adequately powered RCTs is still necessary to confirm any benefit on HF readmission and mortality.

### 4.3. Diuretics

Loop diuretics (furosemide, bumetanide, tor(a)semide) have been used for the treatment of volume overload for decades not only in HF but also in other diseases characterized by fluid retention (e.g., renal disease or venous valve insufficiency) or as antihypertensive agents. Loop diuretics inhibit the Na^+^/K^+^/-2Cl^−^ co-transporter in the thick ascending limb of the Henle loop in the nephron, increasing (Na^+^) excretion and enhancing diuresis [62]. Despite the fact that diuretics are fundamental for the relief of symptoms of congestion in HF, they have various, occasionally severe, side effects [63,64]. These include hypovolemia, electrolyte disturbances, hypersensitivity, and ototoxicity, especially with furosemide [65]. The excessive diuresis due to high loop diuretics doses can induce the contraction of the volume of the extracellular fluid, resulting in contraction metabolic alkalosis (CMA) [66]. The latter, the most notorious disturbance of the acid–base balance in HF, is characterized by HCO_3_^−^ levels exceeding >30 mEq/L, is usually associated with a slight elevation in the serum anion gap [67], and typically develops in the setting of diuretic-induced decongestion. CMA may be present in as many as ≈30–40% of HF patients [68], with some estimates suggesting that the prevalence may reach 47% in acute HF [69]. The mechanisms underlying CMA include chloride loss, diminished effective arterial blood volume, activation of RAAS, increased (Na^+^) delivery to the distal nephron, hypokalemia, and possibly hypochloremia [70]. Further, the treatment of HF patients with loop diuretics may decrease the glomerular filtration rate due to alterations in renal blood flow and glomerular filtration pressure [71]. Finally, of outmost importance is the fact that loop diuretics activate RAAS and SNS, the two fundamental pathways in HF progression, and these side effects must be countered by the co-administration of RAAS inhibitors (angiotensin-converting enzyme inhibitors/angiotensin receptor blockers/angiotensin receptor–neprilysin inhibitors (ARNI)/mineralocorticoid receptor antagonists (MRA)), β-blockers, and sodium–glucose transporter 2 (SGLT2) inhibitors (SGLT2i) [72].

Assessing the effect of diuretics on the outcome in HF is a complex problem, as the separation of the diuretic risk from the negative prognostic effect of needing diuretics because of congestion is not an easy task. In an effort to address this issue, the interaction between loop diuretics and outcome was recently examined in a study analyzing administrative data of nearly 200,000 individuals divided into the following: (i) patients without HF diagnosis treated with loop diuretics; (ii) HF patients not treated with loop diuretics; (iii) HF patients treated with loop diuretics; and (iv) patients without HF diagnosis not using loop diuretics who served as a reference comparator [73]. Five-year mortality was slightly higher for HF patients in the absence of loop diuretics, whereas it was substantially higher (≈40%) among those with diuretics but no HF, and more than twice as high for HF patients treated with loop diuretics compared with patients without loop diuretics or HF diagnosis. The relatively favorable outcome of HF patients not treated with loop diuretics may suggest less severe disease or that the standard HF treatment used during the inclusion period adequately prevented water and salt retention, or both. Whether the use of loop diuretics represents an unnecessary patient risk cannot be concluded from this study, which is based on analyses of administrative datasets, necessitating the conduction of randomized trials of diuretic initiation or withdrawal [74]. However, as it is highly unlikely that such trials will ever be conducted, caution is required when these agents are used in HF patients.

A worrying event during loop diuretic treatment, which often ensues in acute and advanced HF and is accompanied by a rise in morbidity and mortality, is the development of diuretic resistance, the inability to achieve sufficient fluid and (Na^+^) output and consequently decongestion despite progressive increases in diuretic dosage [75]. The most common therapeutic approach to diuretic resistance is the addition of diuretics that block different nephron segments on top of loop diuretics (sequential nephron blockade) [76]. In this regard, there is evidence suggesting that the addition of acetazolamide, which inhibits carbonic anhydrase in the proximal tubule of the nephron and impedes bicarbonate and (Na^+^) reabsorption by interacting with the Sodium/Hydrogen Exchanger 3, results in a higher incidence of successful decongestion vs. loop diuretics alone [77,78]. Likewise, hydrochlorothiazide, which inhibits the (Na^+^)/(Cl^−^) cotransporter in the luminal side of the distal tubule leading to (Na^+^) and water diuresis, added on top of loop diuretic therapy seems to augment the loop diuretic response [79,80]. Finally, there is evidence to suggest that tolvaptan (an antagonist of vasopressin 2 receptor predominantly expressed on the basolateral membrane of the distal convoluting tubule and collecting duct) removes hypotonic fluid that corrects hyponatremia at the same time and is equally effective to oral or intravenous thiazides as an add-on treatment to loop diuretics in patients with diuretic resistance in the setting of decompensated HF [81].

### 4.4. Renal Replacement Therapy (RRT)

Roughly half of hospital-admitted patients with acute HF develop acute renal dysfunction, which may require temporary RRT to support kidney function, especially in cases with diuretic resistance or severe metabolic abnormalities. The optimal timing for the initiation of RRT and the best RRT modality (hemodialysis, ultrafiltration, or peritoneal dialysis) in acute HF are currently controversial [82]. Ultrafiltration, the removal of isotonic plasma water across a semipermeable membrane driven by the application of hydrostatic pressure gradient, has been regarded a promising alternative to intense diuretic therapy, as fluid removal with this treatment modality presents several advantages over diuretics such as the removal of a greater amount of (Na^+^) with less neurohormonal activation [83]. However, the findings of relevant clinical trials are conflicting, emphasizing the necessity of establishing valid patient selection criteria and fluid removal targets.

## 5. Clinical Implications

**Figure 4:**
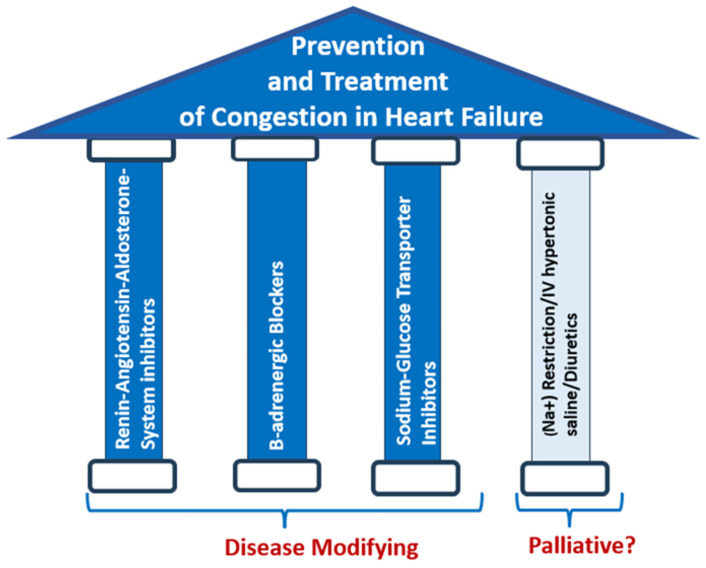
Disease-modifying treatment with renin–angiotensin–aldosterone inhibitors (angiotensin-converting enzyme inhibitors/angiotensin receptor blockers/angiotensin receptor–neprilysin inhibitors/mineralocorticoid receptor antagonists), B-adrenergic blockers, and especially sodium–glucose transporter 2 inhibitors, which additionally inhibit proximal tubule sodium (Na^+^) reabsorption, is the cornerstone for the prevention and treatment of (Na^+^) retention leading to congestion in HF. Interventions directly targeting (Na^+^) such as strict dietary sodium restriction, intravenous hypertonic saline (IV saline), and diuretics should be additionally implemented in selected patients with florid congestion to alleviate symptoms as they are not devoid of adverse effects and their effect on outcome is doubtful.

A dominant event determining the HF course includes the disruption of the delicate balance of (Na^+^) and water leading to (Na^+^) and water retention and edema formation. Although incomplete decongestion has been associated with increased HF morbidity and mortality, interventions directly aiming at (Na^+^) are of doubtful significance in terms of outcome improvement [4]. Regarding loop diuretics, these medications should be judiciously used only in patients with established congestion. Even in these patients, it is of outmost importance, if not previously prescribed, to ensure the initiation and rapid up-titration of quadruple HF therapy with ARNI, β-blocker, MRA, and SGLT2i (“big four”) in order to augment the decongestive effect of diuretics, counter their side effects, and improve the outcomes of HF patients [11,84,85] (Figure 4).

Regarding the diet of HF patients, liberal fruit and vegetable consumption, avoidance of excessive fats, moderate dietary salt restriction (2000–3000 mg/day), and moderate carbohydrate and animal protein intake should be encouraged [27,86,87]. The avoidance of a strict dietary (Na^+^) restriction is further supported by the fact that benefit from the use of IV HSS on top of IV furosemide cannot be excluded [88]. Thus, although the retention and accumulation of (Na^+^) is a characteristic feature of HF, current interventions aimed at directly lowering (Na^+^), such as strict dietary (Na^+^) restriction and the inadvertent or overaggressive use of loop of diuretics, should be discouraged in most HF patients and implemented only to those with acute or advanced, congestive disease.

The possibility that the limited effectiveness of (Na^+^)-targeting treatments is partly due to the fact that the current metrics of HF severity cannot accurately predict and prevent episodes of congestion and guide (Na^+^)-targeting treatments, often leading to dysnatremias not being excluded. In a study that recruited 1000 consecutive patients with HF with a median follow-up time for survivors of 5.1 years, both hyponatremia (*n* = 72) and hypernatraemia (*n* = 98) were associated with a significantly increased mortality and a U-shaped association of serum (Na^+^) with mortality risk was found [89].

Recent evidence suggests that spot urinary sodium (SUS) measurements may be used as a guide to monitor (Na^+^)-targeting interventions. In this regard, the implementation of SUS measurements may predict and consequently prevent episodes of decompensation in chronic HF, whereas SUS measurements at admission and after diuretic treatment have been associated with better decongestion and improved outcomes in acute HF [90]. Further, the traditional (2)-compartment model has been set aside by the (3)-compartment model emphasizing the non-osmotic storage of (Na^+^), especially in the skin. ^23^(Na^+^) MRI enables the accurate and reliable measurement of tissue (Na^+^), and preliminary findings show that elevated skin (Na^+^) content in hypertensive and HF patients is associated with left ventricular remodeling. It is reasonable, therefore, that interventions targeting (Na^+^) should also aim at reducing the increased tissue (Na^+^) content in HF [91].

## 6. Future Directions

Humans show a heterogeneous hemodynamic response to (Na^+^) intake which has long been attributed to variations in renal (Na^+^) handling. However, this traditional (2)-compartment model of (Na^+^) storage has been recently displaced by the (3)-compartment model, emphasizing the importance of the skin as a major non-osmotic regulator of (Na^+^) homeostasis. Accumulating evidence strongly suggests that the local regulatory action of cutaneous blood flow as well as NaCl and water metabolism contribute to cardiovascular hemodynamic control and that their disruption in HF may have detrimental effects. The contemporary developments towards the understanding of the (Na^+^) homeostasis in HF may have potential implications for clinical practice. On this subject, ^23^(Na^+^)-MRI could prove to be a useful tool in guiding decongestive treatment and developing and optimizing novel NaCl-targeting approaches. Tissue (Na^+^) is mobilizable by several interventions such as intravenous furosemide, empagliflozin, and dialysis. ^23^(Na^+^)-MRI may render feasible the identification of patients who might benefit from modified therapeutic approaches to optimize (Na^+^) removal. As the natriuresis levels induced by loop diuretics decrease with repeated dosing, ^23^(Na^+^)-MR imaging may be employed to potentially identify patients suffering from more severe (Na^+^) overload and who might benefit from additional therapy. Moreover, by monitoring (Na^+^) tissue storage with ^23^(Na^+^)-MR imaging, the incidence of dysnatremias with (Na^+^)-targeting therapies in HF might be reduced. A fascinating alternative approach having the potential of widespread applicability is the analysis of human sweat, which contains a multitude of diverse analytes, and the analysis of its composition can inform the state of the body [92]. In recent years, several sweat sensing technologies, including devices that employing ion-selective electrodes to monitor electrolytes such as (Na^+^) and (Cl^–^), have been developed [93,94]. Undoubtably, further studies employing ^23^(Na^+^)-MRI technology and wearable sensors are required to learn more about the clinical significance of tissue (Na^+^) storage and (Na^+^)-related mechanisms of morbidity and mortality in HF.

## 7. Conclusions

Congestion, a key driver of poor outcomes in HF, is due to (Na^+^) and water retention. Interventions directly targeting (Na^+^), such as strict dietary (Na^+^) restriction, intravenous hypertonic saline, and diuretics should be implemented only on top of the “big four” in selected patients with florid congestion in order to alleviate symptoms, as diuretics are not devoid of adverse effects and their impact on outcomes is doubtful. Spot urinary sodium measurements may be used as a guide to monitor (Na^+^)-targeting interventions since they seem to improve their efficacy. Hopefully, monitoring tissue (Na^+^) with ^23^(Na^+^)-MR imaging and possibly the analysis of human sweat, which contains a multitude of diverse analytes, with biosensors may revolutionize the maintenance of (Na^+^) and water balance in HF.

## Data Availability

Not applicable.
